# “We can’t get along without each other”: Qualitative interviews with physicians about device industry representatives, conflict of interest and patient safety

**DOI:** 10.1371/journal.pone.0174934

**Published:** 2017-03-30

**Authors:** Anna R. Gagliardi, Pascale Lehoux, Ariel Ducey, Anthony Easty, Sue Ross, Chaim Bell, Patricia Trbovich, David R. Urbach

**Affiliations:** 1 Toronto General Hospital Research Institute, University Health Network, Toronto, Ontario, Canada; 2 Department of Public Health Administration, University of Montreal, Montreal, Quebec, Canada; 3 Department of Sociology, University of Calgary, Calgary, Alberta, Canada; 4 Institute of Biomaterial & Biomedical Engineering, University of Toronto, Toronto, Ontario, Canada; 5 Women & Children’s Health Research Institute, University of Alberta, Edmonton, Alberta, Canada; 6 Department of General Internal Medicine, Mount Sinai Hospital, Toronto, Ontario, Canada; University of Ottawa, CANADA

## Abstract

**Objectives:**

Physician relationships with device industry representatives have not been previously assessed. This study explored interactions with device industry representatives among physicians who use implantable cardiovascular and orthopedic devices to identify whether conflict of interest (COI) is a concern and how it is managed.

**Design:**

A descriptive qualitative approach was used. Physicians who implant orthopedic and cardiovascular devices were identified in publicly available directories and web sites, and interviewed about their relationships with device industry representatives. Sampling was concurrent with data collection and analysis. Data were analyzed and discussed using constant comparative technique by all members of the research team.

**Results:**

Twenty-two physicians (10 cardiovascular, 12 orthopedic) were interviewed. Ten distinct representative roles were identified: purchasing, training, trouble-shooting, supplying devices, assisting with device assembly and insertion, supporting operating room staff, mitigating liability, conveying information about recalls, and providing direct and indirect financial support. Participants recognized the potential for COI but representatives were present for the majority of implantations. Participants revealed a tension between physicians and representatives that was characterized as “symbiotic”, but required physicians to be vigilant about COI and patient safety, particularly because representatives varied regarding disclosure of device defects. They described a concurrent tension between hospitals, whose policies and business practices were focused on cost-control, and physicians who were required to comply with those policies and use particular devices despite concerns about their safety and effectiveness.

**Conclusions:**

Given the potential for COI and threats to patient safety, further research is needed to establish the clinical implications of the role of, and relationship with device industry representatives; and whether and how hospitals do and should govern interaction with representatives, or support their staff in this regard.

## Introduction

Conflict of interest (COI) has been defined as “a set of circumstances that creates a risk that professional judgment or actions regarding a primary interest will be unduly influenced by a secondary interest” [1,2]. Physicians’ clinical decisions and practice patterns can be influenced by industry relationships [3,4]. The potential for COI may be widespread; according to public registers in the United States, financial relationships with industry were reported by 45% of otolaryngologists, 50% of orthopedic surgeons, 55% of plastic surgeons, 60% of urologists and 78% of neurologists [5].

Various approaches have been implemented by funders, professional societies and academic institutions to manage COI. Disclosure policies are the most commonly applied strategy to address COI [6–8]. However, research shows that guideline authors [9] and professional association meeting participants [10] failed to disclose payments from industry. Analysis of COI policies at American [11] and Canadian [12] universities and research institutions found that, while the majority had COI policies, they varied in criteria for what to disclose and how to manage COI. Limitation is another strategy for managing COI whereby organizations such as hospitals apply policies that prohibit employees from accepting industry incentives, or restrict visits from industry representatives [13]. Despite the presence of such limitation policies, physicians who were interviewed said that they trusted and valued information from drug industry representatives, and felt that it benefited patients [14,15].

Physician relationships with device industry representatives have not been previously studied to identify whether COI is a concern or how it is managed. This is particularly relevant among physicians who use higher-risk cardiovascular or orthopedic devices that have been associated with adverse events [16,17], many of whom are involved in their development, or pre- or post-market evaluation [18,19]. Furthermore, interaction with representatives of the device industry differs from relations with drug industry representatives. Both types of representatives discuss and promote the uses and benefits of their products with physicians but, in contrast to drug representatives, device representatives are frequently present during instances of patient care when their products are being used. In part this occurs because hospitals seek to optimize the efficiency of supply management, standardize surgery and reduce costs through “just-in-time” delivery of medical devices by representatives [20]. Thus, physician and institutional factors may result in considerable interaction between physicians and device representatives. To better understand this phenomenon, including whether physician relations with device representatives creates opportunities for COI and how it is managed, this study explored the nature of interactions with device industry representatives among physicians who use implantable cardiovascular and orthopedic devices to identify opportunities for COI that may influence patient safety.

## Methods

### Approach

In the absence of prior empirical research, qualitative methods are needed to provide a descriptive account that can serve as a point of reference for future research. A descriptive qualitative approach was used [21,22]. This qualitative approach does not test or generate theory. Instead, it is the most commonly used method to gather explicit information about views and experiences when seeking to understand the implications of phenomenon for health care practice or policy. Rigor and transferability were optimized using standard strategies and reporting criteria, for example, independent analysis of data by two or more investigators and inclusion of select quotes from a variety of participants (S1 Table) [23,24]. Ethics approval for this study was granted by the University Health Network Research Ethics Board. There was no relationship with participants, who provided written informed consent prior to scheduling and conducting interviews.

### Sampling and recruitment

The sampling frame included Canadian physicians who use implantable cardiovascular or orthopedic devices because these are higher-risk devices that have been associated with adverse events that impact patient safety [16,17] and many individuals in these specialties are likely involved in device development or evaluation, which may lead to COI [18,19]. Physicians were identified in publicly available certification agency directories, and hospital or university web sites, and invited to participate by regular or electronic mail. They were purposively recruited to ensure representation by specialty (cardiac or vascular surgery, interventional cardiology, orthopedic surgery), region (Canadian provinces), setting (academic, community); and years in practice (self-reported early, mid or late career). A reminder was sent to non-respondents at two and four weeks from initial contact. Sampling in qualitative research is dependent on achieving thematic saturation, however, we initially aimed to interview at least ten physicians with experience in each of cardiovascular or orthopedic implantable devices who also varied by other sampling characteristics. Thematic saturation was determined through discussion among the research team on two occasions upon review of themes and exemplar quotes extracted from interview transcripts.

### Data collection

Telephone interviews were conducted and audio-recorded by the principal investigator between April 8 and September 28, 2015. Interviews averaged 31 minutes. Participants were asked: What is the nature of your relationship with device industry representatives? Subsequent prompts were dependent on participant responses but generally included: How often do you interact with the representative, what support do they provide, how do you manage COI, and what is the hospital policy on representatives and COI?

### Data analysis

All members of the research team identified unique themes inductively using constant comparative technique [21]. First, the principal investigator extracted all useable data from each transcript and applied preliminary codes that represented potential themes. This data were independently reviewed by all members of the research team on two separate occasions during data collection to assess the extent of thematic saturation, and their feedback was used to modify the coding scheme by adding, expanding, editing or merging thematic codes. Once all interviews were completed, the principal investigator again reviewed all transcripts to ensure that all relevant data were extracted. Data were again independently reviewed by the research team and discussed as a group to further refine the coding scheme. Data (quotes organized by thematic codes) were tabulated by theme and summarized (S2 Table). The summary was reviewed and discussed by the research team on a fourth occasion to interpret the data.

## Results

### Participants

Twenty-two physicians who implanted cardiovascular (n = 10) and orthopedic devices (n = 12) were interviewed (Table 1) from among 27 who consented of the 561 that were invited to participate. These included 8, 10 and 4 early, mid and late career physicians, respectively, from five provinces. Views were similar between those who implanted cardiovascular and orthopedic devices.

**Table 1:** Demographic characteristics of interview participants.

Physician specialty	Self-reported career stage			Subtotal
	Early	Mid	Late	
Orthopedic surgeons				
	• 10OCE-MB • 11OTE-MB • 14OTE-AB • 15OTE-NS • 16OTE-NS • 17OTE-NS	• 06OTM-ON • 08OTM-MB • 12OCM-BC	• 03OTL-ON • 07OTL-ON • 09OCL-MB	12
Cardiac or vascular surgeon, or interventional cardiologists	• 02CTE-ON • 04CTE-ON	• 01CTM-ON • 05CTM-ON • 13CTM-MB • 19CTM-ON • 20CTM-AB • 21CTM-ON • 22CTM-MB	• 18CTL-ON	10
Subtotal	8	10	4	22

C cardiac, O orthopedic; T teaching, C community; E early career, M mid-career, L late career; two letter code for province.

### Potential for COI recognized by physicians

A few participants adamantly said that they themselves maintained a distant relationship with representatives to avoid potential industry influence on their choice of devices.

I don’t like having the reps in the room generally. I want the people who are using the equipment to know it and learn it and there’s no encouragement to do that if the people are being spoon fed all the time. I know that there is a common kind of relationship and a sense of chumminess that happens in orthopedics and I want none of it. I have never taken any money from an equipment company directly. I don’t have consulting relationships with any of them. I just won’t do it (06OTM)

I purposely don’t keep a close relationship with them because I don’t want to be influenced by different products from their standpoint. Each company has devices that are best suited for specific reasons. One of the things that I pride myself on is being able to use the correct implants for the deformity. When you develop a relationship sometimes you get yourself caught in a hole where you don’t want to use other implants when sometimes you probably should (14OTE)

### Representatives routinely present

In contrast, most participants described “symbiotic” relationships with representatives and said “we can’t get along without each other” (03OTL), referring to a variety of circumstances during which they interacted with device representatives. Interaction was frequent—participants said that representatives were present in the operating room for the majority of cases: “My rep is there 95% of the time or more” (12OCM) and “They would come to all the implantations” (13CTM). Furthermore, relationships with device representatives were said to be long-standing, suggesting that many representatives were well-known to physicians.

Some of the reps I’ve known for 20 years (20CTM). You see them in the hospital, in the hallway. I’ll actually stop and chat with them and I’ve had coffee or, if you’re at a meeting, dinner with them (20CTM)

These people have been veterans in the industry, they’ve worked for a number of different companies. I’ve seen them through residency and through my training (10OCE)

### Physicians reliant on representatives

Representatives served multiple roles (Table 2). Information and support from representatives was considered an important factor in making individual or group decisions about which devices to purchase. Apart from colleagues, representatives were most frequently mentioned as a source of information about devices. Representatives supported training by either providing or linking physicians with training opportunities, and providing support during the learning curve period. Most notably, representatives were needed to ensure that devices, many of which are complex and comprised of a large number of components, were properly selected, assembled and implanted. By providing support to the operating room nurses, participants said that they could instead focus on the procedure. This was thought to reduce the duration of surgery, enhance patient safety and outcomes, and potentially mitigate liability for an adverse outcome. This was said to be even more important in community settings with few operating room staff.

**Table 2:** Reported roles of medical device industry representatives.

Theme	Exemplary Quote
Purchasing	They’re always available if we want to meet to discuss a product. They’ve been good at saying some people don’t use this, this other one is more simple, so they do keep us posted on what other people use more often (11OTE)
Training	When we bring in a new device there’s usually a lot of support. The rep will be in town when we’re doing the first few procedures. They provide appropriate support in the perioperative period with the team to make sure that people know how to use the interface for the device and so forth. Once that period is over there is often remote contact with the rep who is usually available whenever we need via phone. And if need be they will fly back into town but that’s less frequent as time goes on (22CTM)
Present for surgical procedures	In orthopedics it’s a very close interaction. We can’t get along without each other. Especially the newer systems, they’re so complicated that you need the company representative to help the nurses assemble the implants and pick out the pieces. We see the industry reps all the time (03OTL)
Supply devices when needed	My rep is there for my cases 95% of the time or more. Sometimes I have questions about a design issue with some of their implants. Other times there will be issues with supplies, we don’t have enough of this or that. They’re also there to cycle out implants that are reaching their expiry date, and they’re there to teach the nurses how to use all the stuff (12OCM)
Assembly	There’s a relationship with them in terms of them being there to support their product. They support the nurses in terms of the instrumentation, partially because my experience in using different companies so that sometimes some of the instrumentation is slightly different. Their role is to come and support them to allow me to do the surgeries quicker and safer and not have to worry about the nursing side. So relationships with the reps are important because they’re there to help facilitate ease of the case (14OTE)
Presence mitigates liability	We do tend to use them heavily. For a primary joint replacement you don’t need to have a rep in the room if it’s the standard system that you use day in and day out. The problem is when you have a revision knee system that’s got 13 pans of instrumentation and there are three hundred different ways to assemble the implant based on the different options that are available it’s useful to have the rep in the room. There’s also some shared liability because there are so many different ways to modify and customize the revision implants that to have them say, no that’s not the right one, that actually goes with the next size larger, it’s the one right next to it, you just need to go one more over on the shelf and then you’ll have the right implant. So they help with the inventory and reduce the risk of wrong implants being assembled and inserted then charged to the facility (08OTM)
Beneficial impact on patient care	When you have a really good rep that works with you they can really have a profound impact on patient care because they know instrumentation so well (17OTE)
Information about recalls	The majority of time if there’s any problem with the device usually companies send a note that the device had to be recalled. Those are sent as a notification to each surgeon’s office (01CTM)
Trouble-shooting support	If something adverse occurs we inform the company because we want some guidance or direction on whether that’s something other people have seen, or their recommendation in terms of dealing with a scenario. The company facilitates the ability for us to contact international groups with greater experience to consult (04CTE)
Direct and indirect financial support	Support for academic or research activities. That’s usually in the form of supporting journal clubs or research endeavours (02CTE)

### Hospital policies may create opportunities for COI

Participants noted that their hospitals no longer purchased or stocked medical devices. Instead, representatives delivered devices to the operating room when needed. This represents a hospital policy that encourages physician-representative interaction.

We figure out the size or length or whatever it is we need and then he picks it off his selection. It’s like looking at a menu. They actually carry in the instruments to do the operation because the hospital doesn’t own them (07OTL)

Participants said that they were required to use devices from preferred vendors based on hospital or regional purchasing contracts, which further required physicians to interact with specific representatives. Participants said that this often resulted in disgruntled physicians, surgical complications, poor patient outcomes, and increased rather than reduced costs.

The group purchases that orthopedics has been involved in, things like cautery machines, staplers, have all been horrible and we’ve gone back to what we were using before. It’s just a disaster. They save the money and then it’s just horrible. It was like a mass protest. I think the group purchase was done without real consultation. Now it seems to be more participatory but it seems like we’re still purchasing things that most of the surgeons don’t like (10OCE)

We’ve had experience that if you force surgeons to change implants based on a contract that your complication rate goes up for a while. So it makes good business sense until you actually go and look at your revision costs over the next months to two years and then, all of a sudden, all of your cost-savings went into pain and suffering of patients and their subsequent care (08OTM)

In contrast to these policies that encouraged physician-representative interaction during instances of patient care, and preferential use of devices that were not considered ideal for patients or physicians, one participant referred to a hospital-level policy or practice of limiting interaction with representatives.

In our hospital we purposely stay away from any gathering, partying, conferencing, or accepting anything from the manufacturer because that’s unethical (18CTL)

### Physician responsibility to manage COI and protect patient safety

Given the indispensable role of representatives, participants said it was their own responsibility to manage potential COI.

The main thing is to disassociate any sense of obligation toward the rep. I don’t take any money from them and, if they all have equal access, then there’s no way you can be biased. So I have a good relationship with them. But it’s definitely not at arms-length because when I have issues I want to be able to approach them and complain (20CTM)

Participants said that representatives varied regarding disclosure of device warnings. In such cases physicians verified information or changed products to ensure patient safety.

There are a number of other companies we use where the representation falls well below the standard and it’s well-known. In relationships like that your guard is up, you’re always double-checking everything, you’re always verifying with other colleagues that have used that system to make sure that you’re doing the best you can for the patient (10OCE)

If we ever got into a situation where I felt that they were putting patients at risk I would just go crazy on them. For example, there was a problem with a type of wire that we use in coronaries where the manufacturer knew there was a problem and left them on the shelves. So I basically stopped using any of their stuff (21CTM)

## Discussion

Physicians who use implantable devices recognized that relationships with device industry representatives created the potential for COI to influence decision-making about devices, and for patient safety to be compromised if representatives did not share information about device warnings. However, they assumed responsibility for managing such relationships because representatives function as indispensable members of the operating room team with specialized and proprietary information that may be essential to achieving optimal patient outcomes. To manage COI, physicians said they avoided industry representatives, avoided taking money from them, consulted with colleagues for advice about devices, or stopped using a particular device if they had concerns about it. Hospital policies and business practices may encourage COI by facilitating representative presence in the operating room and, through purchasing contracts, mandating the use of particular devices that, in the view of physicians, were not safe or effective. Overall this research revealed a tension between physicians and device representatives that was characterized as “symbiotic”, but required physicians to be vigilant about COI and patient safety. There was a distinct concurrent tension between hospitals, whose policies and business practice were focused on cost-control, and physicians who were required to comply with those policies and practices, and use particular devices despite concerns about their safety and effectiveness.

Strengths of this study included the use of rigorous qualitative methods to capture detailed information about the physician-device representative relationship. Sampling was purposive to capture a variety of perspectives from individuals with differing characteristics, and achieved thematic saturation which signals that recruitment was sufficient to thoroughly identify relevant themes. However, several limitations should be mentioned. The study involved a small number of participants, and they were sampled from Canadian hospitals. Therefore the findings may not be transferrable to other settings, and should be confirmed through investigation of these themes in other settings.

Few comparable studies were identified, and largely focused on drug industry gifts or research funding. American physicians who took part in focus groups [14] and a survey [25] considered interactions with, and gifts and funding from drug representatives as positive and beneficial, disagreed that such relationships affected physician behaviour, and rationalized that they remained impartial. Similarly, physicians in this study said that device representatives offered many beneficial services and they could manage COI. Studies of academic institutions in the United States [11] and Canada [12] found that policies concerning COI were absent in hospitals and, as a result, the onus was on individual physicians to self-regulate. This also appeared to be true in our study. While there are similarities between representatives of drug companies and device companies there are also differences–drugs are not stocked in a just-in-time manner, may be much simpler to use, do not require hands-on instruction, and may not be subject to the same types of contractual restrictions compared with devices. Future research should explore whether these differences lead to different types of physician-industry interaction with differing implications for COI. Given that physicians may not have the capacity to objectively judge their own performance [26], ongoing research is warranted to assess the impact of behavioral interventions that influence cognitive dissonance, or awareness of actual versus desirable behavior, such as audit and feedback [27] on views about COI and relationships with industry representatives.

Participants in our study noted the variable reliability of representatives, which raises questions about the characteristics of such individuals. A study by Mueller et al. involving focus groups with 17 American cardiovascular device industry representatives reported that they had former experience in nursing, pharmacy, engineering, business, veterinary medicine and administration [28]. Notably, the same study revealed that device representatives experienced role conflicts and moral distress regarding their activities in the clinical setting and customer service obligations [28]. Representatives said that physicians and nurses lacked knowledge about devices, and asked them for assistance beyond their expected duties including making clinical decisions about how devices should be programmed and managed, deactivating devices, and informing patients and family about the deactivation process and outcomes. They said that their customer service responsibilities had grown tremendously in recent years, and referred to this as an unpaid service to remedy staffing shortages in the clinical setting. They were reluctant to decline clinician requests, fearing that this would affect their relationship with clinicians and impact their ability to sell their companies’ products. The study by Mueller et al. suggests the presence of a third tension between clinicians and device representatives that further contributes to the potential for COI. Ongoing research involving device representatives should further investigate the challenges they experience when interacting with physicians that may compromise patient safety. Such research could also involve nurses who may be witness to industry representative-physician interactions in the operating room.

These findings are novel because there is little research on the implications of representative presence in the operating room. In response to concern about their role and influence, some hospitals have instituted policies specifying that representatives must assist medical staff only verbally, acquire a distinct identification badge on each visit and, prior to their first visit, provide documentation of competency in the specific device, training in infection control, insurance certificate, proof of annual tuberculosis test and signed confidentiality agreement [29]. Nursing and surgeon professional societies have issued similar statements regarding the credentialing and appropriate role of representatives [30,31]. A survey of senior nurses in charge of 79 gynecology operating theatres in the United Kingdom found that 82% had no guidelines for representative presence in the operating room, and 42% obtained patient consent for visits [32]. In surveys of arthroplasty patients in Canada and the United States, most respondents reported that their physician had not mentioned COI in advance of surgery [33–36]. Those patients trusted physicians to self-regulate, and believed that oversight should be provided by professional associations. Current professional society standards for COI focus on how their own organization manages COI, for example, funding of continuing education and annual meetings, rather than how they can help individual members to mitigate COI in their daily interactions with industry representatives [37–39].

In conclusion, this exploratory study identified tensions between physicians, hospitals and device industry representatives that may contribute to COI. Physicians may be reliant on industry representatives for their knowledge and skill, and hospitals, by employing purchasing agreements and just-in-time operating room delivery of devices, promote physician reliance on device industry representatives. Further research is needed to identify the clinical implications of the characteristics and role of, and relationship with medical device industry representatives from the physician, nurse and representative perspective; how that relationship and its implications differ from physician interaction with drug industry representatives; whether and how hospitals do and should govern interaction with medical device representatives; and explore the views of leadership from professional societies on how they could help their members manage COI, and the effectiveness of behavioral interventions that influence physician views about COI.

## Supporting information

S1 Table COREQ checklist for reporting qualitative research. (DOCX)

S2 Table Data on role of device industry representatives. (DOCX)
