# Supplementary material for: “We can’t get along without each other”: Qualitative interviews with physicians about device industry representatives, conflict of interest and patient safety
Source: PLoS One. 2017 Mar 30;12(3):e0174934. doi: 10.1371/journal.pone.0174934 (PMC5373623; doi:10.1371/journal.pone.0174934)
Supplement: S2 Table — (DOCX) [file pone.0174934.s002.docx]

S2 Table. Data on role of device industry representatives

| Provide information about a product and underlying evidence to support purchase decisions | - You ask a rep to come and give a presentation to the entire division about this particular product (01CTM) - The company reps are the ones that provide us with the data. The company rep would say here’s device A and here is the published data on device A. They will tell us here’s a cohort study of 300 patients who had this valve and everything went well for the duration of the study. Would you be interested in using this and adding to the clinical experience and performing your own evaluation (05CTM) | - There is a lot of information directly from the manufacturers. They have to give you all their information about legal proceedings and class actions. I have been able to get that (07OTL) - They’re always available if we want to meet to discuss a product. They’ve been good at saying some people don’t use this, this other one is more simple, so they do keep us posted on what other people use more often and they will tell me (11OTE) |
| --- | --- | --- |
| Provide clinical support (on assembly, use of devices, stock devices, replace defective devices) | - The first priority we have is patient care and support for clinical activities. If there was any issue somebody would be there to visit or you’d be on the phone. Clinical support includes teaching nurses and other health care practitioners how to use and take care of them (02CTE) - They provide support in the operating room in assembling the devices and ensuring that we’re putting them in correctly. We consult with them regarding difficult patients and whether people around the world have had experience with them for instance. The company facilitates the ability for us to contact international groups with greater experience to consult (04CTE) - They would have a representative in our city that would be very familiar with the technology. They would come to all the implantations. For our complex devices, heart failure devices and defibrillators, those we generally have representatives from companies with us (13CTM) - In my experience in three hospitals, one in Canada and two in the United States, we call them technologists from the manufacturer, would be there to train nurses and doctors prior to using that device, and sometimes but not always present when the device is being used for technical issues (18CTL) - Routinely (present during cases). But the primary responsibility is with the physician. We may ask questions and have a dialogue about things but the ultimate responsibility is that of the physician. I think that you can benefit from the experience of the representatives. They are not there to push you to use additional product or to buy any products, and they do have technical knowledge that can be helpful during the course of conducting a case (19CTM) - There’s people with experience that I speak with beforehand on the telephone and discuss cases with other surgeons (20CTM) | - In orthopedics it’s a very close interaction. We can’t get along without each other. Especially the newer systems, they’re so complicated that you need the company representative to help the nurses assemble the implants and pick out the pieces. We see the industry reps all the time (03OTL) - For joint implants there’s usually an instrument rep. Or when we’re using a special fracture device often there is an instrument rep (07OTL) - It depends on the company, it can be quite variable. It could also depend a little bit on the rep and how good they are at communicating back. Some reps are better than others. Some companies are better than others (06OTM) - We do tend to use them heavily, especially for our revisions. And it’s as much for the nursing staff as it is for us. For a primary joint replacement you don’t need to have a rep in the room if it’s the standard system that you use day in and day out because the nurses are fine with it. The problem is when you have a revision knee system that’s got 13 pans of instrumentation and there are three hundred different ways to assemble the implant based on the different options that are available with a revision implant it’s useful to have the rep in the room (08OTM) - I’m in a community hospital, we don’t have an orthopedic team, it’s a general team that’s doing everything. So having the reps around is invaluable (10OCE) - I do hip and knee replacements and my rep is there for my cases 95% of the time or more. If it’s a device that I’m not familiar with or the nurses aren’t familiar with the reps are there a lot. Sometimes I have questions about a design issue with some of their implants. Other times there will be issues with supplied, we don’t have enough of this or that. They’re also there to cycle out implants that are reaching their expiry date and they’re there to teach the nurses how to use all the stuff (12OCM) - [regarding who the reps are] Their background is variable. The guy that does our joint replacements, he’s a civil engineer. His predecessor brought him to a bunch of cases and taught him all the information about implants and, in fact, I helped train him a little bit when I was in the operating room (12OCM) - There’s a relationship with them in terms of them being there to support their product. They support the nurses in terms of the instrumentation, partially because my experience in using different companies so that sometimes some of the instrumentation is slightly different. Their role is to come and support them to allow me to do the surgeries quicker and safer and not have to worry about the nursing side. So relationships with the reps are important because they’re there to help facilitate ease of the case (14OTE) - We have a couple of different vendors here. For the devices I use, representation is probably present for 75% of the cases. Their main role for me is to make sure all the technical aspects of their product are understood by the entire team because there are such a large number of pieces to each set of equipment, to understand what each of the devices are and how they work together from a technical point of view with that specific set of instrumentation (15OTE) - They are present for every case. They are there for information about what implants are available or not and they usually have experience with the implant (16OTE) - When you have a really good rep that works with you they can really have a profound impact on patient care because they know instrumentation so well. Despite the fact that they’ll say maybe you should try this and maybe you should try that, ultimately you always have the final decision, and it’s nice to think about other suggestions tat we should potentially try (17OTE) |
| Shared liability for wrong revision |  | - There’s also some shared liability in wrong implant opening because there are so many different ways to modify and customize the revision implants that to have them say, no that’s not the right one, that actually goes with the next size larger, it’s the one right next to it, you just need to go one more over on the shelf and then you’ll have the right implant. So they help with the inventory and reduce the risk of wrong implants being assembled and inserted then charged to the facility (08OTM) |
| Financial support | Support for academic or research activities. That’s usually in the form of supporting journal clubs or research endeavours (02CTE) | Orthopedic surgeons are involved in some of the design. I’ve never been involved in design and I have no royalties in anything. I do not get paid by any company for speaking engagements. So from that point of view I’m neutral (09OCL) |
| Conflict of interest | - In our hospital we purposely stay away from any gathering, partying, conferencing, or accepting anything from the manufacturer because that’s unethical. If you go to a conference we have to state if there’s an interest or association, or if funding came from the manufacturer (18CTL) - I have a very good relationship with all reps. That even extends to people that I don’t even use their device. Some of the reps I’ve known for 20 years. I’ve never put some of their devices in but you still see them in the hospital, in the hallway. I’ll actually stop and chat with them and I’ve had coffee, or if you’re at a meeting, dinner with them (20CTM) - The main thing is to disassociate any sense of obligation toward the rep. They all have equal access to me. I don’t take any money from them and, if they all have equal access, then I don’t see any value. There’s no way you can be biased. So I have a good relationship with them. But it’s definitely not at arms-length because when I have issues I want to be able to approach them and complain (20CTM) | - Many orthopedic surgeons are involved with implant development and by and large it’s a symbiotic relationship. There is of course a lot of marketing and competition. So you have to declare all that, if you’re paid to be a consultant or if you’ve got stock in the company. And there’s a great many orthopedic surgeons, including myself, whose children end up involved in the industry because you sort of group up with it. They either work for the companies, they’re engineers or sales people or marketing people. So you have to declare that as a conflict (03OTL) - You can go to the industry but the industry is biased. Ultimately you have to maintain that impartiality (10OCE) - I don’t have a close relationship with any of them. I have a friendly relationship with them. I purposely don’t keep a close relationship with them because I don’t want to be influenced by different products from their standpoint. Each company has devices that are best suited for specific reasons. One of the things that I pride myself on is being able to use the correct implants for the deformity. When you develop a relationship sometimes you get yourself caught in a hole where you don’t want to use other implants when sometimes you probably should of if you’re too comfortable with one implant only that may not be the correct one to put in (14OTE) |
| Variable relationship with reps | - They’re definitely not present in the operating room unless it’s a new device. Any time we have a new device they do train, they are present in the OR, they do have an orientation to the device. Either there will be a proctor from another site, a physician, or else the rep who has significant exposure being present (20CTM) | - We don’t have that relationship with our reps at all. Maybe one case every two weeks I would have a rep there. It would be a specific request either around an implant that we don’t use very often or something particularly challenging around a particular case. I don’t like having the reps in the room generally. I want the people who are using the equipment to know it and learn it and there’s no encouragement to do that if the people are being spoon fed all the time (06OTM) - I don’t like a too close relationship with the reps. I do not want to be somebody’s boy. I think I’m a real outlier in the orthopedic community, it’s not who I am. I know that that’s a common kind of relationship and a sense of chumminess that happens in orthopedics and I want none of it. I’m just not interested in it (06OTM) - I have never taken any money from an equipment company directly. I just don’t do it. I don’t have consulting relationships with any of them. I just won’t do it (06OTM) - At the same time we take an oath as a doctor but sometimes we get chastised because it’s wrong if a guy buys you a cup of coffee. I have a problem with that. I think we all have to be our own police. I probably have some of the best reps. These people have been veterans in the industry, they’ve worked for a number of different companies and I’m lucky because their mantra is honesty and service. I’ve seen them through residency and through my training and they’ve not once changed their tune. There’ve been incidents where I’ll come to them and say, this is the problem, do you have an implant that can do this, and the standard is ‘yup, we got that, but this company does it better. So that’s the relationship that I am working with. There’s a number of other companies we use that the representation falls well below the standard and it’s well known. In relationships like that your guard is up, you’re always double-checking everything, you’re always verifying with other colleagues that have used that system to make sure that you’re doing the best you can for the patient (10OCE) - It happens in less than 10% of my cases that they come in with a product in the OR, someone is there with me if I’m using something new that I have not used that often (11OTE) - I wouldn’t say that I have as close a relationship with them that some other people do because in Peds we use less fancy implants. We just put in K wires and the children heal nicely so it’s less of a big market for them (11OTE) |
